# Fungal Endophytic Communities of Two Wild *Rosa* Varieties With Different Powdery Mildew Susceptibilities

**DOI:** 10.3389/fmicb.2018.02462

**Published:** 2018-10-16

**Authors:** Yi Zhao, Zhi Xiong, Guangli Wu, Weixiao Bai, Zhengqing Zhu, Yonghan Gao, Shobhika Parmar, Vijay K. Sharma, Haiyan Li

**Affiliations:** Medical School of Kunming University of Science and Technology, Kunming, China

**Keywords:** wild rose, powdery mildew, fungal endophytic community, disease resistance, Illumina MiSeq

## Abstract

Powdery mildew (PM) is one of the most devastating and wide spread fungal diseases of rose, which seriously decrease its productivity and commercial value. In the present study, the endophytic fungal communities of two wild *Rosa* varieties (*Rosa multiflora* Thunb and *R. multiflora* var. *carnea* Redouté and Thory) with different PM susceptibilities were studied through Illumina MiSeq sequencer. A total of 14,000,424 raw reads were obtained from 60 samples, and 6,862,953 tags were produced after merging paired-end reads. 4462 distinct OTUs were generated at a 97% similarity level. It was found that only 34.2% of OTUs shared between two plant varieties. All of the OTUs were assigned into four fungal phyla, 17 classes, 43 orders, 86 families, 157 genera, and 208 species. Members of Ascomycota were found to be the most common fungal endophytes (EF) among all plant samples (93.7% relative abundance), followed by Basidiomycota (4.7% relative abundance), while Zygomycota and Glomeromycota were found to be rare and incidental. At each developmental stage of plants, the diversity and community structure of EF between two *Rosa* varieties showed significant differences. Both PCoA plots and PERMANOVA analyses indicated that developmental stage was the major factor contributing to the difference between the *Rosa* varieties (*R*^2^ = 0.348, *p* < 0.001). In addition, plant varieties and tissues were also important factors contributing to the difference (*R*^2^ = 0.031, *p* < 0.05; *R*^2^ = 0.029, *p* < 0.05). Moreover, *Neofusicoccum, Rhodosporidium*, and *Podosphaera*, etc., were found to be significantly different between two *Rosa* varieties, and some of the endophytes may play a role in PM resistance. These finding are encouraging to testify the potential use of these fungi in the biocontrol of PM in future studies.

## Introduction

Roses are one of the most economic and important ornamental crops (Debener and Byrne, 2014), however, they are adversely affected by some fungal pathogens. Powdery mildew (PM) is one of the most devastating and widespread fungal diseases of rose, which is mainly caused by *Podosphaera pannosa* (Wallr.) de Bary (Belanger et al., 1994; Braun and Takamatsu, 2000). The pathogen appears on the upper surface of young leaves in the form of white powdery pustules which ultimately cover the entire leaf surface, thus resulting lower yield, poor market value and consumer acceptance. The use of various fungicides has been the main control strategy adopted against the disease (Pasini et al., 1997; Kim et al., 2008; Debener and Byrne, 2014). However, the decreasing efficacy of many fungicides as well as the risk of fungicide residues on the leaves and flowers has highlighted the need for more effective and safe alternative control measures. One of the potential methods of reducing the severity of PM in an environmentally safe manner is the use of biocontrol agents. Some isolates of *Ampelomyces quisqualis* Ces., *Lecanicillium lecanii* Zimm., *Bacillus subtilis, Bacillus velezensis* CC09, and *Gjaerumia minor* Nyland have been found to be effective biocontrol agents as PM control (Nasir et al., 2014; Mmbaga et al., 2016; Cai et al., 2017).

Endophytic fungi (EF) can be defined as fungi that reside asymptomatically in the interior of host plant tissues (Hyde and Soytong, 2008). They have been widely studied and found to be ubiquitous within all examined plants. They play an important role in biotic and abiotic stress tolerance of host plants (Li et al., 2012b; O’Hanlon et al., 2012; Ibrahim et al., 2017). Burketova et al. (2015) demonstrated that induced resistance can be conferred by plant-associated microorganisms. O’Keeffe et al. (2017) also pointed out that plant-associated microbes influenced the emergence, spread and evolution of plant pathogens. Therefore, EF can be explored as potential biocontrol agents. Despite the increasing interest in EF, little is known about the diversity and community structure of EF associated with *Rosa* spp. as well as its ecological roles. Bahig et al. (2012) studied the culturable endophytic bacterial community of rose plants inhabiting dry desert ecosystems. They found that some endophytic bacteria were able to produce indole acetic acid (IAA), soluble phosphate and siderophore. Bibi et al. (2012) also used culture-dependent method to study the endophytic bacterial community of *Rosa rugosa* Thunb and found that some isolates exhibited *in vitro* inhibitory activity against target plant pathogenic oomycetes.

Although *Rosa multiflora* Thunb (RSM) is a wild *Rosa* species highly resistant to PM, *R. multiflora* var. *carnea* Redouté and Thory (RSMC) is a variant of *R. multiflora* is highly susceptible to PM (Wylie, 1954; Chatani et al., 1996; Leus, 2005; Zhang et al., 2009; Qiu et al., 2015). Fungal endophytic communities of *Rosa* are still poorly known. Thus, present study was designed to examine fungal endophytic communities at different developmental stages of these two *Rosa* varieties having variable susceptibilities to PM.

The recent advanced cutting edge technologies such as high-throughput sequencing technology have now enabled us to study the microbial colonization under specific environmental conditions and habitat in unprecedented details (Beeck et al., 2015; Eevers et al., 2016). These high-throughput sequencing techniques can be used to detect both culturable and unculturable microorganisms, and thus can reflect the diversity and community structure more close to its natural state (Eevers et al., 2016). Here we report the first comprehensive investigation of the fungal endophytic communities associated with different varieties of *R. multiflora* through Illumina sequencing technology.

## Materials and Methods

### Sample Collection

The plants of RSM and RSMC were collected from the Cangshan Mountain, Dali, Yunnan Province, Southwest China (25°25′–27°58′ N, 99°58′–100°27′ E) on the 15^th^ of each month between April and August, 2015. This timing was decided considering different developmental stages of PM in rose at the sampling location, i.e., April was the month of early stage, May and June were the major outbreak period, while July and August were the period of later stage (Leus, 2005; Xiang et al., 2017). The altitudes of sampling sites were between 1,980 and 2,220 m a.s.l. Sampling and further analysis were done in triplicate. Each replicate consisted of 15 branches from three plants of corresponding variety of *Rosa*. All samples were cut down with sterile scissors, placed in a sterile plastic bag and transported to the lab and processed within 24 h.

### Total Genomic DNA Extraction and Sequencing

The plants were separated as leaves and stems, and then washed in running tap water and processed as follows: the samples were cut into segments and surface-sterilized by sequentially dipping into 75% ethanol (2 min) and 5% sodium hypochlorite (2 min), then, washed with sterile distilled water and dried on sterile filter paper (Li et al., 2012a). The efficiency of the surface sterilization process was confirmed by making imprints of disinfected plant fragments on Petri dishes containing PDA (potato dextrose agar); the absence of any fungal growth was observed as an effective surface sterilization (Schulz et al., 1998). Afterwards, the surface-sterilized tissues were homogenized in sterile mortars with liquid nitrogen. The total genomic DNA was extracted by PowerSoil^®^ DNA Isolation Kit (Mobio, United States), and then was verified by gel electrophoresis (1% agarose, 120 V, 30 min). The fragment of the ITS2 region (200–400 bp) was targeted using the primers ITS3_KYO2 (5′-GATGAAGAACGYAGYRAA-3′) and ITS4 (5′-TCCTCCGCTTATTGATATGC-3′) for fungal community analysis (White et al., 1990; Bokulich and Mills, 2013). PCR reactions were performed in a 25 μl volume and contained: 2.5 μl 10 × PCR buffer, 1.5 μl Mg^2+^ (25 mM MgCl_2_), 2.5 μl dNTP mixture (4 mM each), 0.5 μl KOD-Plus-Neo (1 units/μl; TOYOBO), 1 μl Template DNA (0.4 ng), 2.5 μl primer (10 μM each), and 14.5 μl sterilized double-distilled H_2_O. The PCR program consisted of an initial denaturation step at 94°C for 5 min, followed by 30 cycles of denaturation at 94°C for 20 s, annealing at 50°C for 30 s, elongation at 72°C for 30 s, with a final extension of 5 min at 72°C. The PCR products were purified with an OMEGA Gel Extraction Kit (Omega Bio-Tek, United States) according to the manufacturer’s protocol. The library quality was assessed on the Qubit 2.0 Fluorometer (Thermo Scientific) and Agilent Bioanalyzer 2100 system. Paired-end sequencing (2 × 250 bp) was carried out on an Illumina MiSeq sequencer at Rhonin Biosciences Co., Ltd. (Chengdu, China). The Illumina sequencing data obtained in these experiments are publicly available in the NCBI Sequence Read Archive under No. SRR5312521.

### Analysis of Pyrosequencing Data

The raw pyrosequencing data were obtained in FASTQ files along with sequencing quality files. Paired-end reads from the original DNA fragments theoretically were merged using FLASH, which is a very fast and accurate analysis tool (Magoč and Salzberg, 2011). All sequences were denoised, as well as trimmed for barcodes and primers. The cleaned-up sequences were aligned and classified with those in the SILVA rRNA database. Chimeric sequences, as well as mitochondrial and chloroplast sequences were removed using UCHIME (Edgar, 2013). The remaining sequences were subsampled (excluding singletons) and grouped into operational taxonomic units (OTUs) based on a 97% similarity criterion (Eevers et al., 2016). Taxonomy were assigned using the UNITE database^1^. Rarefaction curves were performed to check the sample adequacy using a 50 sequence increment. The representative sequence for each OTU was provided taxonomical annotation by a naive Bayesian classifier, the Ribosomal Database Project (RDP) Classifier^2^ at 0.5 confidence threshold (Wang et al., 2007).

### Statistical Analysis

The diversity indices and rarefaction curves were performed with R software (Liaw and Wiener, 2002). In addition, the difference of Shannon diversity index (*H*′) between two different groups was analyzed by one-way ANOVA (Response variable residuals are normally distributed and variances of populations are equal) or Kruskal–Wallis test in SPSS (Yatsunenko et al., 2012). To calculate the relative abundance of each OTU per sample pool, matching reads were divided by the total number of processed reads in the sample pool (Turnbaugh et al., 2009). Bray–Curtis dissimilarities were calculated using the “vegdist” function of the R package using measurable OTU abundances (Coleman-Derr et al., 2016). Venn diagrams were plotted with the package “Venn Diagram.” Principal coordinate analysis (PCoA) based on Bray–Curtis dissimilarities were used to examine community dissimilarity and determine the impact of experimental factors on microbial community structure (Forsberg et al., 2014). To find out the differences among groups, permutational ANOVAs (PERMANOVAs) were performed with the function “adonis” in the package “vegan” (Desgarennes et al., 2014). PERMANOVAs were used to elucidate the contribution of following factors to the dissimilarity among the endophytic fungal communities: plant varieties, plant tissues (leaf and stem), and developmental stages (Peršoh, 2013; Pérez-Izquierdo et al., 2017).

## Results

### Sequencing Yields

**FIGURE 1:**
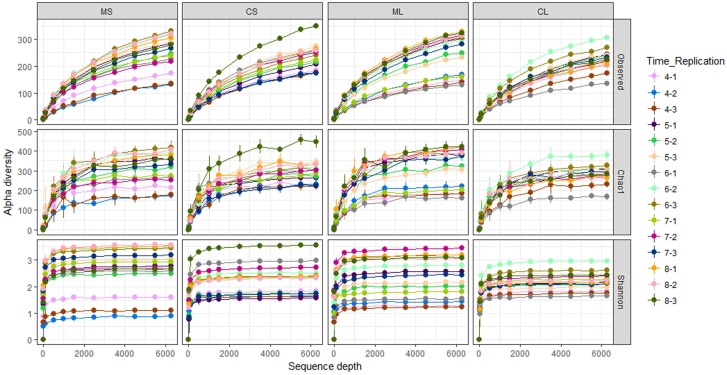
Rarefaction curves of the observed OTU number, Chao1, and Shannon index at 97% similarity of samples. CL and CS, ML and MS: leaf and stem of *R. multiflora* var. *carnea* and *R. multiflora*, respectively; 1, 2, and 3 represent triplicate; 4, 5, 6, 7, and 8 stand for the sampling time April, May, June, July, and August.

A total of 14,000,424 raw reads were obtained from the sequencer, and 6,862,953 tags were produced after merging paired-end reads (length > 200 bp, without ambiguous base “N,” and average base quality score > 30), the average length was 340 base pairs. Excluding all reads that showed no clear taxonomic affinity, 4462 distinct OTUs were generated at a 97% similarity level (**Table 1**). The rarefaction curves of all the plant samples tended to approach the saturation plateau (**Figure 1**).

**Table 1:** The detailed information of tags and number of OTUs from different samples.

Sample	Raw PE	Raw tags	Clean tags	Effective tags	Number of OTUs
C-L	4421416	2167361	1321247	1321237	1840
C-S	3379208	1656474	680511	680508	1907
M-L	3480020	1705892	1207530	1207523	2031
M-S	2719780	1333226	811236	811228	2029


*C-L and C-S, M-L and M-S: Leaf and stem of *R. multiflora* var. *carnea* (RSMC) and *R. multiflora* (RSM), respectively.*

The OTUs were assigned into four fungal phyla, 17 classes, 43 orders, 86 families, 157 genera, and 208 species. Members of Ascomycota were found to be the most common EF among all plant samples (93.7% relative abundance), followed by Basidiomycota (4.7% relative abundance), while Zygomycota and Glomeromycota were found to be rare and incidental (**Supplementary Figure S1**).

**FIGURE 2:**
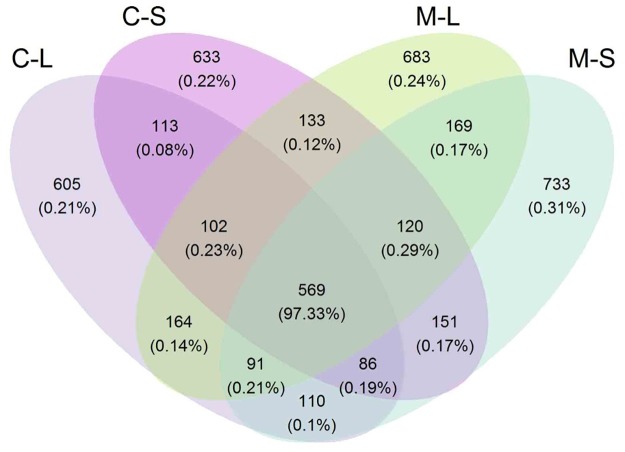
Venn diagram describing the OTU distribution across two *R. multiflora* varieties and their tissues. CL and CS, ML and MS: leaf and stem of *R. multiflora* var. *carnea* and *R. multiflora*, respectively.

The Venn diagram illustrates the distribution of fungal communities across samples (**Figure 2**). It was found that 34.2% OTUs found in RSM (both leaf and stem) shared in RSMC. On the contrary, 35.5 and 30.3% OTUs were only detected in RSM and RSMC, respectively. The OTUs of RSM (both leaf and stem) were higher than that of RSMC (**Figure 2** and **Table 1**).

### Diversity of Fungal Endophytes

**FIGURE 3:**
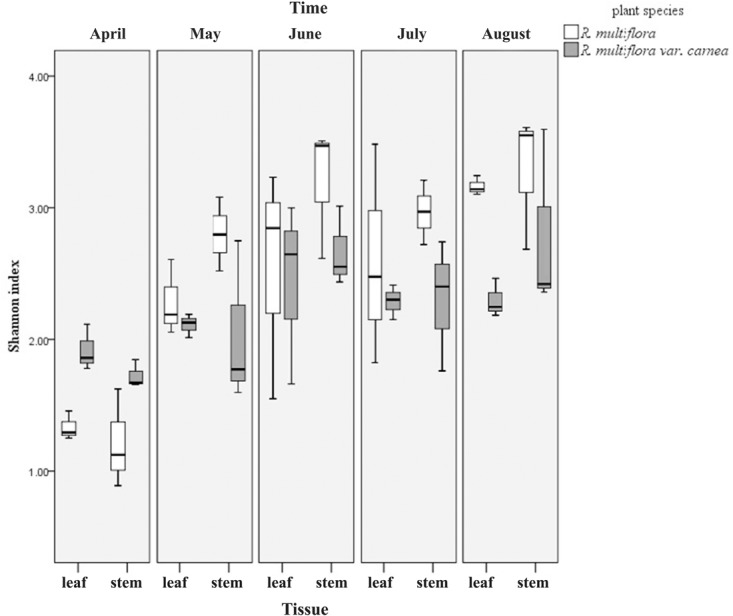
Shannon index (*H*′) of endophytic fungi from two *R. multiflora* varieties at different developing stages, demonstrated by box plots with median and 95% confidence intervals displayed.

Computational analyses of the Shannon index (*H*′) estimated the richness and evenness of EF associated with plant tissues at OTU cutoffs of 0.03 distance units (Klaubauf et al., 2010; Glynou et al., 2016; **Figure 3** and **Supplementary Table S1**). Remarkably, the *H*′ of both plant varieties increased with the development of PM. However, at the early stage (April), the *H*′ of EF of RSM was significantly lower than that of RSMC at the tissue level. But contrary to this, at the middle and later stages (May–August), the *H*′ of RSM was higher than that of RSMC, although the difference was not significant (**Supplementary Table S1**). In addition, it was found that at the same developmental stage, the *H*′ between leaves and stems of the same plant species showed no significant difference (one-way ANOVA, *p* > 0.05).

Rarefaction curves of *H*′ showed that the diversity of EF of RSM fluctuated substantially during the developmental stages, ranging from 1.3 to 3.5 (leaves) and 0.9 to 3.6 (stems), respectively. However, the diversity of EF of RSMC did not fluctuate so greatly when compared with RSM, ranging from 1.7 to 3.0 (leaves) and 1.6 to 3.6 (stems). Similarly, rarefaction curves of the observed OTUs and the Chao1 estimator of EF of RSM fluctuated largely than that of RSMC (**Figure 1**).

### Fungal Endophytic Community Composition

**FIGURE 4:**
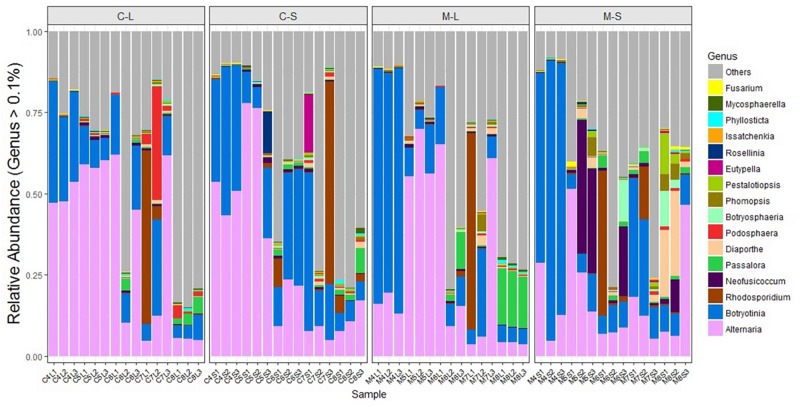
Relative abundance of endophytic fungi of two *Rosa* varieties at genus-level. The fungi with a relative abundance below 0.1% were grouped as “others.” CL and CS, ML and MS: leaf and stem of *R. multiflora* var. *carnea* and *R. multiflora*, respectively; 1, 2, and 3 represent triplicate samples; 4, 5, 6, 7, and 8 stand for the sampling time April, May, June, July, and August.

Fungal endophytic communities of the two *Rosa* varieties showed a certain difference at each developing stage. At the early stage (April), although *Alternaria* and *Botryotinia* were the dominant genera of both rose varieties (**Figure 4** and **Table 2**), while their average relative abundance showed significant differences (one-way ANOVA, *p* < 0.05). In PM resistant rose variety (RSM), *Botryotinia* was the most dominant genus and its average relative abundance was more than 71% both in leaves and stems. However, in PM susceptible rose variety (RSMC), its average relative abundance was lower than 39%, both in leaves and stems (**Table 2**). On the contrary, *Alternaria* was the most dominant genus of RSMC, and the average relative abundance was more than 49%.

**Table 2:** Dominant endophytes associated with two *Rosa* varieties at different developmental stages.

Time	Dominant genera			
	
	*R. multiflora*		*R. multiflora* var. *carnea*	
		
	Leaf	Stem	Leaf	Stem
April	*Botryotinia* (71.83%)	*Botryotinia* (74.07%)	*Alternaria* (49.38%)	*Alternaria* (49.19%)
	*Alternaria* (16.16%)	*Alternaria* (15.29%)	*Botryotinia* (30.37%)	*Botryotinia* (38.66%)
May	*Alternaria* (60.47%)	*Alternaria* (30.33%)	*Alternaria* (58.95%)	*Alternaria* (63.43%)
	*Botryotinia* (9.88%)	*Neofusicoccum* (24.77%)	*Botryotinia* (9.18%)	*Botryotinia* (12.62%)
June	*Alternaria* (29.94%)	*Rhodosporidium* (15.64%)	*Alternaria* (39.05%)	*Botryotinia* (26.97%)
	*Botryotinia* (11.14%)		*Botryotinia* (15.76%)	*Alternaria* (18.09%)
July	*Alternaria* (23.54%)	*Botryotinia* (25.25%)	*Alternaria* (26.33%)	*Botryotinia* (25.51%)
	*Rhodosporidium* (20.28%)	*Alternaria* (11.94%)	*Rhodosporidium* (19.20%)	*Rhodosporidium* (21.04%)
	*Botryotinia* (12.70%)		*Botryotinia* (15.61%)	
			*Podosphaera* (13.25%)	
August	*Passalora* (16.84%)	*Alternaria* (20.04%)		*Alternaria* (11.85%)
		*Diaporthe* (16.23%)		


*Data represents the average of total relative abundance, the fungi with a relative abundance below 10.0% were not shown in the table.*

In May, *Alternaria* remained to be the dominant genus of both rose varieties. Strikingly, *Neofusicoccum* was detected at a higher level in the stems of RSM (average relative abundance of 24.77%) when compared with that (1.09%) of RSMC (**Supplementary Figure S2**). While, the relative abundance of *Neofusicoccum* was almost same in the leaves of both plant varieties (**Supplementary Figure S2**). In June, *Rhodosporidium* was found to be the most dominant genus in the stem of RSM, while *Botryotinia* was the most dominant genus in the stem of RSMC, followed by *Alternaria*.

In July, *Rhodosporidium* was detected in all samples, and it showed a dominant presence in both rose varieties (**Table 2**). Remarkably, *Podosphaera*, the causal fungus of PM in rose, was detected in RSMC with higher relative abundance (13.25%), but was almost absent (0.62%) in RSM (**Figure 4** and **Table 2**). In August, *Passalora* and *Diaporthe* were found to be the dominant genera respectively in the leaves and stems of RSM, while *Alternaria* was the only dominant genus in RSMC.

### Factors Driving Endophytic Fungal Communities

**FIGURE 5:**
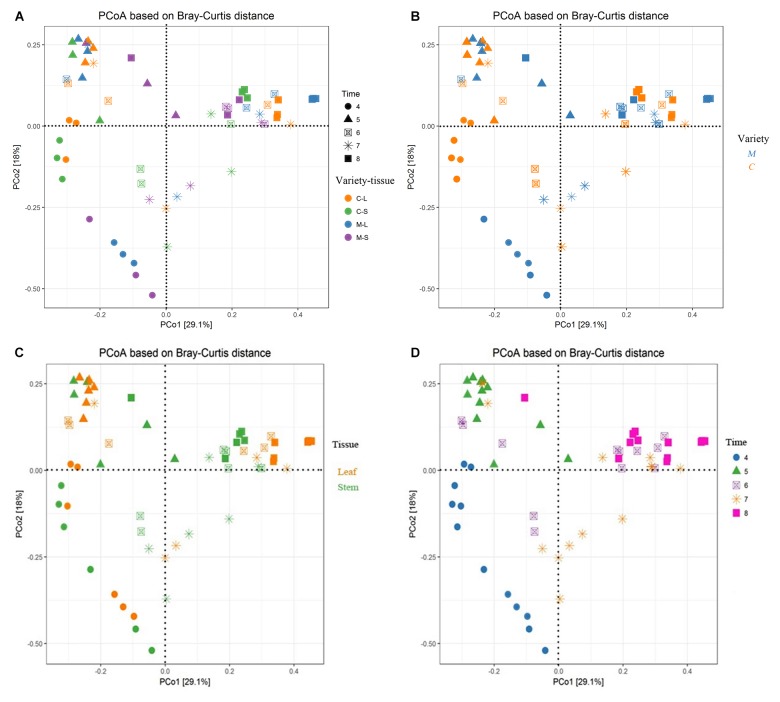
Main factors driving the endophytic microbiota composition of two *Rosa* varieties. Principal coordinate analysis (PCoA) among all sixty samples based on Bray–Curtis distance matrixes. **(A)** All factors; **(B)** plant varieties factor; **(C)** plant tissue factor; and **(D)** developmental stage factor. CL and CS, ML and MS: leaf and stem of *R. multiflora* var. *carnea* and *R. multiflora*, respectively; 4, 5, 6, 7, and 8 denotes the sampling time April, May, June, July, and August.

Permutational ANOVA analysis indicated that the developmental stage was the major factor contributing to the difference between the endophytic fungal communities of two *Rosa* varieties (*R*^2^ = 0.348, *p* < 0.001). In addition, plant varieties and tissues were also important factors contributing to the difference (*R*^2^ = 0.031, *p* < 0.05; *R*^2^ = 0.029, *p* < 0.05) (**Supplementary Table S2**). The results were supported by PCoA using the Bray–Curtis dissimilarity matrixes on rarefied OTUs to identify the main drivers of microbial composition. PCoA plots displayed clustering of plant varieties, plant tissues and developmental stages, but the relative contribution of each factor differed. The samples from RSM in April were grouped together and clustered separately as compared to the other samples (**Figure 5A**). Similarly, in May, the stem samples of RSM differed comparably from the samples collected in other months (**Figure 5A**). In order to further differentiate the variables contributing to the distribution of endophytic fungal community between two wild rose varieties, we evaluated the impact of plant variety, plant tissue and developmental stage, separately (**Figures 5B–D**). Regardless of plant varieties and plant tissues, with the exception of June and July, the samples were grouped into distinct clusters according to the developmental stage (**Figure 5D**). In other words, our data indicate that endophytic fungal communities of wild roses were more influenced by developmental stage, whereas the plant varieties and tissues played a minor role in shaping the microbiome of the studied rose varieties.

## Discussion

A total of 4462 distinct OTUs were generated from two rose varieties at a 97% similarity level. The OTUs were assigned into four fungal phyla. Ascomycota was found to be the most common EF among the plant samples (93.7% relative abundance). This is consistent with previous findings that Ascomycota was the dominant group of EF in many plant species from various environments (Gazis and Chaverri, 2010; Peršoh et al., 2010; Khan et al., 2017). *Alternaria* and *Botryotinia* are very common and have been reported as the dominant EF of various plant species (González and Tello, 2011; Setati et al., 2015). In the present study also, they were found to be the most dominant EF (**Table 2**). Similarly, *Alternaria* was found to be the dominant EF of *R. rugosa* and *R. hybrid* in our previous studies (Zhou et al., 2014). Moreover, Kumar and Chandel (2018) found that the phylloplane fungus *Alternaria* sp. from rose can inhibit conidial germination of *P. pannosa in vitro*.

Endophytic fungal community differs across plant species, soil type, climate, altitude, and other environmental conditions (Hardoim et al., 2008; Lundberg et al., 2012; Siddique and Unterseher, 2016; Asemaninejad et al., 2018). In the present study, samples of RSM and RSMC were collected at each developmental stage parallel from the same site. However, their endophytic fungal communities were significantly different, and there were only 34.2% of the OTUs shared between them (**Figures 1–3** and **Supplementary Table S1**). We suggest that the difference mainly come from the difference of plant varieties. PERMANOVA analysis also supported this suggestion. Plant varieties contributed more to shape the endophytic community than the other factors (**Supplementary Table S2**). The result was consistent with previous findings that host plant species can influence the diversity and composition of microbial endophytes (Inceoğlu et al., 2010; Ding et al., 2013; Hardoim et al., 2015). Raghavendra et al. (2017) also found that *Mimosa pigra* L. (Fabaceae) had similar fungal communities within stems among regions, suggesting that host plant rather than environment determined endophytic communities in this species.

Siddique and Unterseher (2016) found that the microbiota was little affected by the physiological state of the leaves. Lundberg et al. (2012) also found that the developmental stage of plants contributed less to shape the community composition. However, in the present study, both PCoA plots and PERMANOVA analyses indicated that the developmental stage was the major factor contributing the difference between two wild rose varieties. Giordano et al. (2009) also demonstrated that the EF inhabiting sapwood of Scots pine (*Pinus sylvestris* L.) are significantly affected by the degree of decline of trees. We suppose that the pathogen infection can influence the fungal endophytic community. Similarly, Pan et al. (2008) found that the pathogen *Ustilago maydis* DC infection had significant effect to the endophytic community of maize (*Zea mays* L.). Moler and Aho (2018) also found that the pathogen infection affected white bark pine foliar fungal endophytic community. In addition, it was demonstrated that the plant tissue also have important effects on the fungal community (**Figures 5A,C** and **Supplementary Table S2**). The same phenomenon was found by Yang et al. (2017). They found plant tissues contribute to shape the bacterial communities associated with tree peony. The influence may be attributed to the endophytic specificity and adaptation to the anatomical aspects and nutritional conditions of different plant organ compartments (Gaiero et al., 2013; Liotti et al., 2018).

Although the *H*′ of both rose varieties increased with PM development (from April to August), while at the early stage of PM (April), the *H*′ of RSM (PM resistant) was significantly lower than that of RSMC (PM susceptible) (**Figure 3** and **Supplementary Table S1**). The same phenomenon was observed by Martin et al. (2013). They found that the resistant plant genotypes exhibit a lower frequency and diversity of fungal endophytes in the xylem of the elm (*Ulmus* spp.) than susceptible plant genotypes. Similarly, Reiter et al. (2002) also found a higher diversity of bacterial endophytes in potato due to the presence of the pathogen *Erwinia carotovora* subsp. *atroseptica*. However, with the development of PM (at the middle and late stages of PM), the *H*′ of RSM was higher than that of RSMC (**Figure 3** and **Supplementary Table S1**). This could be due to the developed defense system of RSM that allows fungal endophytes to colonize in order to suppress the pathogen stress. This hypothetical mechanism needs further investigation.

In addition to fungal endophytic community and diversity, it was found that the relative abundance of some fungal endophytes were significantly different between the two *Rosa* varieties: in May, *Neofusicoccum* spp. was common in the stems of RSM. On the contrary, it was less common in the stems of RSMC (**Supplementary Figure S2** and **Table 2**). *Neofusicoccum batangarum* have been reported as an endophyte of *Terminalia catappa* L. (Shetty et al., 2011). This fungus can produce a diverse variety of phytotoxins that confer high flexibility to adapt several environmental conditions (Abou-Mansour et al., 2015). Therefore, the fungus may have facilitated host plants resistance to PM by secreting phytotoxins. In June, *Rhodosporidium* spp. was found to be the most dominant EF in RSM, while *Botryotinia* spp. was the most dominant EF in RSMC. Contrary to this, *Podosphaera* spp. was detected in higher relative abundance in RSMC (13.25%), however, it was almost absent in RSM (0.62%). *Rhodotorula paludigena* has been reported as an antagonistic yeast, that mainly control postharvest fungal pathogens through the production of lytic enzymes, induction of resistance, formation of biofilms, and competition for limiting nutrients and space (Spadaro and Droby, 2016; Sun et al., 2018). Mmbaga et al. (2008) reported that *Rhodosporidium* spp. were highly effective biological control agents of PM on flowering dogwoods (*Cornus florida* L.). Sansone et al. (2018) have demonstrated that *Rhodosporidiobolus fluvialis* could be used as a biocontrol agent against *Botrytis cinerea*. Therefore, higher endophytic colonization of *Rhodosporidium* spp. may have improved its resistance to PM, and resulted lower *Podosphaera* infestation.

Genotype of the host plant plays a significant role in the selection of associated fungal community (Preto et al., 2017; Kumar and Chandel, 2018), which in turn have a positive or negative effect on host plants (Heijden and Hartmann, 2016). In the present study, two rose varieties have significantly different PM resistance, preferentially harbor some selected EF during different developmental stages that may have important implications on the PM susceptibility. However, to prove this hypothesis, more work need to be carried out in the future.

## Data Availability

The Illumina sequencing data obtained in this study are publicly available in the NCBI Sequence Read Archive under No. SRR5312521.

## Author Contributions

YZ, SP, VKS, and HL designed the research and wrote the paper. YZ, ZX, GW, WB, ZZ, and YG performed the research. YZ, SP, and HL contributed new reagents and analytic tools. YZ, ZX, GW, SP, and VKS analyzed the data.

## Conflict of Interest Statement

The authors declare that the research was conducted in the absence of any commercial or financial relationships that could be construed as a potential conflict of interest.

## Footnotes

1 https://unite.ut.ee/

2 http://rdp.cme.msu.edu/classifier/classifier.jsp

## References

[B1] Abou-Mansour E. Debieux J. L. Ramirez-Suero M. Benard-Gellon M. Magnin-Robert M. Spagnolo A. (2015). Phytotoxic metabolites from *Neofusicoccum parvum*, a pathogen of Botryosphaeria dieback of grapevine. *Phytochemistry* 115 207–215. 10.1016/j.phytochem.2015.01.012 25747381

[B2] Asemaninejad A. Thorn R. G. Branfireun B. A. Lindo Z. (2018). Climate change favours specific fungal communities in boreal peatlands. *Soil Biol. Biochem.* 120 28–36. 10.1016/j.soilbio.2018.01.029

[B3] Bahig E. Salih B. Youssuf G. Hesham E. (2012). Characterization of endophytic bacteria associated with rose plant (*Rosa damascena trigintipeta*) during flowering stage and their plant growth promoting traits. *J. Plant Interact.* 7 248–253. 10.1080/17429145.2011.637161

[B4] Beeck M. O. D. Ruytinx J. Smits M. M. Vangronsveld J. Colpaert J. V. Rineau F. (2015). Below ground fungal communities in pioneer scots pine stands growing on heavy metal polluted and non-polluted soils. *Soil Biol. Biochem.* 86 58–66. 10.1016/j.soilbio.2015.03.007

[B5] Belanger R. R. Labbe C. Jarvis W. R. (1994). Commercial-scale control of rose powdery mildew with a fungal antagonist. *Plant Dis.* 78 420–424. 10.1094/PD-78-0420

[B6] Bibi F. Yasir M. Song G. C. Lee S. Y. Chung Y. R. (2012). Diversity and characterization of endophytic bacteria associated with tidal flat plants and their antagonistic effects on oomycetous plant pathogens. *Plant Pathol. J.* 28 20–31. 10.5423/ppj.oa.06.2011.0123

[B7] Bokulich N. A. Mills D. A. (2013). Improved selection of internal transcribed spacer-specific primers enables quantitative, ultra-high-throughput profiling of fungal communities. *Appl. Environ. Microbiol.* 79 2519–2526. 10.1128/AEM.03870-12 23377949 PMC3623200

[B8] Braun U. Takamatsu S. (2000). Phylogeny of Erysiphe, Microsphaera, Uncinula (Erysipheae) and Cystotheca, Podosphaera, Sphaerotheca (Cystotheceae) inferred from rDNA ITS sequences-some taxonomic consequences. *Schlechtendalia* 4 1–33.

[B9] Burketova L. Trda L. Ott P. G. Valentova O. (2015). Bio-based resistance inducers for sustainable plant protection against pathogens. *Biotechnol. Adv.* 33 994–1004. 10.1016/j.biotechadv.2015.01.004 25617476

[B10] Cai X. C. Liu C. H. Wang B. T. Xue Y. R. (2017). Genomic and metabolic traits endow Bacillus velezensis CC09 with a potential biocontrol agent in control of wheat powdery mildew disease. *Microbiol. Res.* 196 89–94. 10.1016/j.micres.2016.12.007 28164794

[B11] Chatani K. Toyoda H. Ogata Y. Koreeda K. Yoshida K. Matsuda Y. (1996). Evaluation of resistance of rose cultivars and wild rose to powdery mildew and black spot. *Jpn. J. Phytopathol.* 62 202–206. 10.3186/jjphytopath.62.202

[B12] Coleman-Derr D. Desgarennes D. Fonseca-Garcia C. Gross S. Clingenpeel S. Woyke T. (2016). Plant compartment and biogeography affect microbiome composition in cultivated and native *Agave* species. *New Phytol.* 209 798–811. 10.1111/nph.13697 26467257 PMC5057366

[B13] Debener T. Byrne D. H. (2014). Disease resistance breeding in rose: current status and potential of biotechnological tools. *Plant Sci.* 228 107–117. 10.1016/j.plantsci.2014.04.005 25438791

[B14] Desgarennes D. Garrido E. Torres-Gomez M. J. Pena-Cabriales J. J. Partida-Martinez L. P. (2014). Diazotrophic potential among bacterial communities associated with wild and cultivated *Agave* species. *FEMS Microbiol. Ecol.* 90 844–857. 10.1111/1574-6941.12438 25314594

[B15] Ding T. Palmer M. W. Melcher U. (2013). Community terminal restriction fragment length polymorphisms reveal insights into the diversity and dynamics of leaf endophytic bacteria. *BMC Microbiol.* 13:1. 10.1186/1471-2180-13-1 23286760 PMC3546043

[B16] Edgar R. C. (2013). UPARSE: highly accurate OTU sequences from microbial amplicon reads. *Nat. Methods* 10 996–998. 10.1038/nmeth.2604 23955772

[B17] Eevers N. Beckers B. Op de Beeck M. White J. C. Vangronsveld J. Weyens N. (2016). Comparison between cultivated and total bacterial communities associated with *Cucurbita pepo* using cultivation-dependent techniques and 454 pyrosequencing. *Syst. Appl. Microbiol.* 39 58–66. 10.1016/j.syapm.2015.11.001 26656884

[B18] Forsberg K. J. Patel S. Gibson M. K. Lauber C. L. Knight R. Fierer N. (2014). Bacterial phylogeny structures soil resistomes across habitats. *Nature* 509 612–616. 10.1038/nature13377 24847883 PMC4079543

[B19] Gaiero J. R. McCall C. A. Thompson K. A. Day N. J. Best A. S. Dunfield K. E. (2013). Inside the root microbiome: bacterial root endophytes and plant growth promotion. *Am. J. Bot.* 100 1738–1750. 10.3732/ajb.1200572 23935113

[B20] Gazis R. Chaverri P. (2010). Diversity of fungal endophytes in leaves and stems of wild rubber trees (Hevea brasiliensis) in Peru. *Fungal Ecol.* 3 240–254. 10.1016/j.funeco.2009.12.001

[B21] Giordano L. Gonthier P. Varese G. C. Miserere L. Nicolotti G. (2009). Mycobiota inhabiting sapwood of healthy and declining Scots pine (*Pinus sylvestris* L.) trees in the Alps. *Fungal Divers.* 38 69–83.

[B22] Glynou K. Ali T. Buch A. K. Haghi Kia S. Ploch S. Xia X. (2016). The local environment determines the assembly of root endophytic fungi at a continental scale. *Environ. Microbiol.* 18 2418–2434. 10.1111/1462-2920.13112 26530450

[B23] González V. Tello M. L. (2011). The endophytic mycota associated with Vitis vinifera in central Spain. *Fungal Divers.* 47 29–42. 10.1007/s13225-010-0073-x

[B24] Hardoim P. R. van Overbeek L. S. Berg G. Pirttila A. M. Compant S. Campisano A. (2015). The hidden world within plants: ecological and evolutionary considerations for defining functioning of microbial endophytes. *Microbiol. Mol. Biol. Rev.* 79 293–320. 10.1128/MMBR.00050-14 26136581 PMC4488371

[B25] Hardoim P. R. van Overbeek L. S. van Elsas J. D. (2008). Properties of bacterial endophytes and their proposed role in plant growth. *Trends Microbiol.* 16 463–471. 10.1016/j.tim.2008.07.008 18789693

[B26] Heijden M. G. A. Hartmann M. (2016). Networking in the plant microbiome. *PLoS Biol.* 14:e1002378. 10.1371/journal.pbio.1002378 26871440 PMC4752285

[B27] Hyde K. D. Soytong K. (2008). The fungal endophyte dilemma. *Fungal Divers.* 33 163–173.

[B28] Ibrahim A. Sorensen D. Jenkins H. A. Ejim L. Capretta A. Sumarah M. W. (2017). Epoxynemanione A, nemanifuranones A-F, and nemanilactones A-C, from *Nemania serpens*, an endophytic fungus isolated from Riesling grapevines. *Phytochemistry* 140 16–26. 10.1016/j.phytochem.2017.04.009 28441516

[B29] Inceoğlu O. Salles J. F. van Overbeek L. van Elsas J. D. (2010). Effects of plant genotype and growth stage on the betaproteobacterial communities associated with different potato cultivars in two fields. *Appl. Environ. Microbiol.* 76 3675–3684. 10.1128/AEM.00040-10 20363788 PMC2876460

[B30] Khan A. R. Waqas M. Ullah I. Khan A. L. Khan M. A. Lee I.-J. (2017). Culturable endophytic fungal diversity in the cadmium hyperaccumulator *Solanum nigrum* L. and their role in enhancing phytoremediation. *Environ. Exp. Bot.* 135 126–135. 10.1016/j.envexpbot.2016.03.005

[B31] Kim H. S. Kang H. S. Chu G. J. Byun H. S. (2008). Antifungal effectiveness of nanosilver colloid against rose powdery mildew in greenhouses. *Solid State Phenomena* 135 15–18. 10.4028/www.scientific.net/ssp.135.15

[B32] Klaubauf S. Inselsbacher E. Zechmeister-Boltenstern S. Wanek W. Gottsberger R. Strauss J. (2010). Molecular diversity of fungal communities in agricultural soils from Lower Austria. *Fungal Divers.* 44 65–75. 10.1007/s13225-010-0053-1 23794962 PMC3688302

[B33] Kumar V. Chandel S. (2018). Phylloplane microflora diversity of rose and mycoparasitism over rose powdery mildew (*Podosphaera pannosa* (Wallr.) de Bary). *J. Crop Weed* 14 224–229.

[B34] Leus L. (2005). *Resistance Breeding for Powdery Mildew (Podosphaera pannosa) and Black spot (Diplocarpon rosae) in Roses.* Ph.D. thesis, Ghent University, Ghent.

[B35] Li H. Y. Shen M. Zhou Z. P. Li T. Wei Y. L. Lin L. B. (2012a). Diversity and cold adaptation of endophytic fungi from five dominant plant species collected from the Baima Snow Mountain, Southwest China. *Fungal Divers.* 54 79–86. 10.1007/s13225-012-0153-1

[B36] Li H. Y. Wei D. Q. Shen M. Zhou Z. P. (2012b). Endophytes and their role in phytoremediation. *Fungal Divers.* 54 11–18. 10.1007/s13225-012-0165-x

[B37] Liaw A. Wiener M. (2002). Classification and regression by random Forest. *R News* 2 18–22.

[B38] Liotti R. G. da Silva Figueiredo M. I. da Silva G. F. de Mendonca E. A. F. Soares M. A. (2018). Diversity of cultivable bacterial endophytes in *Paullinia cupana* and their potential for plant growth promotion and phytopathogen control. *Microbiol. Res.* 207 8–18. 10.1016/j.micres.2017.10.011 29458872

[B39] Lundberg D. S. Lebeis S. L. Paredes S. H. Yourstone S. Gehring J. Malfatti S. (2012). Defining the core *Arabidopsis thaliana* root microbiome. *Nature* 488 86–90. 10.1038/nature11237 22859206 PMC4074413

[B40] Magoč T. Salzberg S. L. (2011). FLASH: fast length adjustment of short reads to improve genome assemblies. *Bioinformatics* 27 2957–2963. 10.1093/bioinformatics/btr507 21903629 PMC3198573

[B41] Martin J. A. Witzell J. Blumenstein K. Rozpedowska E. Helander M. Sieber T. N. (2013). Resistance to dutch elm disease reduces presence of xylem endophytic fungi in elms (Ulmus spp.). *PLoS One.* 8:e56987. 10.1371/journal.pone.0056987 23468900 PMC3585289

[B42] Mmbaga M. T. Mrema F. A. Mackasmiel L. Rotich E. (2016). Effect of bacteria isolates in powdery mildew control in flowering dogwoods (*Cornus florida* L.). *Crop Prot.* 89 51–57. 10.1016/j.cropro.2016.06.011

[B43] Mmbaga M. T. Sauvé R. J. Mrema F. A. (2008). Identification of microorganisms for biological control of powdery mildew in Cornus florida. *Biol. Control* 44 67–72. 10.1016/j.biocontrol.2007.10.018

[B44] Moler E. R. V. Aho K. (2018). Whitebark pine foliar fungal endophyte communities in the southern Cascade Range, USA: host mycobiomes and white pine blister rust. *Fungal Ecol.* 33 104–114. 10.1016/j.funeco.2018.02.003

[B45] Nasir M. Mughal S. M. Mukhtar T. Awan M. Z. (2014). Powdery mildew of mango: a review of ecology, biology, epidemiology and management. *Crop Prot.* 64 19–26. 10.1016/j.cropro.2014.06.003

[B46] O’Hanlon K. A. Knorr K. Jørgensen L. N. Nicolaisen M. Boelt B. (2012). Exploring the potential of symbiotic fungal endophytes in cereal disease suppression. *Biol. Control* 63 69–78. 10.1016/j.biocontrol.2012.08.007

[B47] O’Keeffe K. R. Carbone I. Jones C. D. Mitchell C. E. (2017). Plastic potential: how the phenotypes and adaptations of pathogens are influenced by microbial interactions within plants. *Curr. Opin. Plant Biol.* 38 78–83. 10.1016/j.pbi.2017.04.014 28505582

[B48] Pan J. J. Baumgarten A. M. May G. (2008). Effects of host plant environment and Ustilago maydis infection on the fungal endophyte community of maize (Zea mays). *New Phytol.* 178 147–156. 10.1111/j.1469-8137.2007.02350.x 18194146

[B49] Pasini C. D’Aquila F. Curir P. Guliino M. L. (1997). Effectiveness of antifungal compounds against rose powdery mildew (*Sphaerotheca pannosa* var. *rosae*) in glasshouses. *Crop Prot.* 16 251–256. 10.1016/S0261-2194(96)00095-6

[B50] Pérez-Izquierdo L. Zabal-Aguirre M. Flores-Renteria D. Gonzalez-Martinez S. C. Buee M. Rincon A. (2017). Functional outcomes of fungal community shifts driven by tree genotype and spatial-temporal factors in Mediterranean pine forests. *Environ. Microbiol.* 19 1639–1652. 10.1111/1462-2920.13690 28181376

[B51] Peršoh D. (2013). Factors shaping community structure of endophytic fungi–evidence from the Pinus-Viscum-system. *Fungal Divers.* 60 55–69. 10.1007/s13225-013-0225-x

[B52] Peršoh D. Melcher M. Flessa F. Rambold G. (2010). First fungal community analyses of endophytic ascomycetes associated with *Viscum album* ssp. austriacum and its host Pinus sylvestris. *Fungal Biol.* 114 585–596. 10.1016/j.funbio.2010.04.009 20943170

[B53] Preto G. Martins F. Pereira J. A. Baptista P. (2017). Fungal community in olive fruits of cultivars with different susceptibilities to anthracnose and selection of isolates to be used as biocontrol agents. *Biol. Control* 110 1–9. 10.1016/j.biocontrol.2017.03.011

[B54] Qiu X. Q. Jian H. Y. Wang Q. G. Zhou N. N. Chen M. Zhang H. (2015). Powdery mildew resistance identification of wild *Rosa* germplasms. *Acta Hortic.* 1064 329–335. 10.17660/ActaHortic.2015.1064.41

[B55] Raghavendra A. K. H. Bissett A. B. Thrall P. H. Morin L. Steinrucken T. V. Galea V. J. (2017). Characterisation of above-ground endophytic and soil fungal communities associated with dieback-affected and healthy plants in five exotic invasive species. *Fungal Ecol.* 26 114–124. 10.1016/j.funeco.2017.01.003

[B56] Reiter B. Pfeifer U. Schwab H. Sessitsch A. (2002). Response of endophytic bacterial communities in potato plants to infection with *Erwinia carotovora* subsp. *atroseptica*. *Appl. Environ. Microbiol.* 68 2261–2268. 10.1128/AEM.68.5.2261-2268.2002 11976096 PMC127529

[B57] Sansone G. Lambrese Y. Calvente V. Fernández G. Benuzzi D. Sanz Ferramola M. (2018). Evaluation of *Rhodosporidium fluviale* as biocontrol agent against *Botrytis cinerea* on apple fruit. *Lett. Appl. Microbiol.* 66 455–461. 10.1111/lam.12872 29495073

[B58] Schulz B. Guske S. Dammann U. Boyle C. (1998). Endophyte-host interactions. II. defining symbiosis of the endophyte-host interaction. *Symbiosis* 25 213–227.

[B59] Setati M. E. Jacobson D. Bauer F. F. (2015). Sequence-based analysis of the *Vitis vinifera* L. cv cabernet sauvignon grape must mycobiome in three south African vineyards employing distinct agronomic systems. *Front. Microbiol.* 6:1358. 10.3389/fmicb.2015.01358 26648930 PMC4663253

[B60] Shetty K. G. Minnis A. M. Rossman A. Y. Jayachandran K. (2011). The Brazilian peppertree seed-borne pathogen, Neofusicoccum batangarum, a potential biocontrol agent. *Biol. Control* 56 91–97. 10.1016/j.biocontrol.2010.09.016

[B61] Siddique A. B. Unterseher M. (2016). A cost-effective and efficient strategy for Illumina sequencing of fungal communities: a case study of beech endophytes identified elevation as main explanatory factor for diversity and community composition. *Fungal Ecol.* 20 175–185. 10.1016/j.funeco.2015.12.009

[B62] Spadaro D. Droby S. (2016). Development of biocontrol products for postharvest diseases of fruit: the importance of elucidating the mechanisms of action of yeast antagonists. *Trends Food Sci. Tech.* 47 39–49. 10.1016/j.tifs.2015.11.003

[B63] Sun C. Fu D. Lu H. Zhang J. Zheng X. Yu T. (2018). Autoclaved yeast enhances the resistance against *Penicillium expansum* in postharvest pear fruit and its possible mechanisms of action. *Biol. Control* 119 51–58. 10.1016/j.biocontrol.2018.01.01

[B64] Turnbaugh P. J. Hamady M. Yatsunenko T. Cantarel B. L. Duncan A. Ley R. E. (2009). A core gut microbiome in obese and lean twins. *Nature* 457 480–484. 10.1038/nature07540 19043404 PMC2677729

[B65] Wang Q. Garrity G. M. Tiedje J. M. Cole J. R. (2007). Naive bayesian classifier for rapid assignment of rrna sequences into the new bacterial taxonomy. *Appl. Environ. Microbiol.* 73 5261–5267. 10.1128/aem.00062-07 17586664 PMC1950982

[B66] White T. J. Bruns T. Lee S. Taylor J. (1990). “Amplification and direct sequencing of fungal ribosomal RNA genes for phylogenetics,” in *PCR Protocols: A Guide to Methods and Application*, eds Innis M. A. Gelfand D. H. Sninsky J. J. White T. J. (SanDiego, CA: Academic Press Inc), 315–322.

[B67] Wylie A. P. (1954). The history of garden roses. *J. R. Hortic. Soc.* 79 555–571.

[B68] Xiang G. S. Zhang Z. J. Wang Q. G. Jian H. Y. Yan H. J. Tang K. X. (2017). Research progress of Chinese rose powdery mildew and its resistance. *Jiangsu Agric. Sci.* 45 9–15.

[B69] Yang R. Liu P. Ye W. (2017). Illumina-based analysis of endophytic bacterial diversity of tree peony (*Paeonia* Sect. *Moutan*) roots and leaves. *Braz. J. Microbiol.* 48 695–705. 10.1016/j.bjm.2017.02.009 28606427 PMC5628320

[B70] Yatsunenko T. Rey F. E. Manary M. J. Trehan I. Dominguez-Bello M. G. Contreras M. (2012). Human gut microbiome viewed across age and geography. *Nature* 486 222–227. 10.1038/nature11053 22699611 PMC3376388

[B71] Zhang H. Yang X. M. Wang J. H. Qu S. P. Li S. F. Tang K. X. (2009). Leaf disc assays of resistance of some Rosa germplasms to the powdery mildew in Yunnan. *Plant Prot.* 25 131–133.

[B72] Zhou Z. P. Zhang C. F. Zhou W. N. Li W. Chu L. Yan J. P. (2014). Diversity and plant growth-promoting ability of endophytic fungi from the five flower plant species collected from Yunnan, Southwest China. *J. Plant Interact.* 9 585–591. 10.1080/17429145.2013.873959

