# Supplementary material for: Fungal Endophytic Communities of Two Wild Rosa Varieties With Different Powdery Mildew Susceptibilities
Source: Front Microbiol. 2018 Oct 16;9:2462. doi: 10.3389/fmicb.2018.02462 (PMC6198141; doi:10.3389/fmicb.2018.02462)
Supplement: Supplementary file 1 [file Data_Sheet_1.doc]

Supplementary Material

# Fungal endophytic communities of two wild *Rosa* varieties with different powdery mildew susceptibilities

Yi Zhao, Zhi Xiong, Guangli Wu, Weixiao Bai, Zhengqing Zhu, Yonghan Gao, Shobhika Parmar, Vijay K. Sharma, Haiyan Li^*^

*** Correspondence:** Prof. Haiyan Li, E-mail: lhyxrn@163.com; Tel.: +86 (871) 5920751; Fax: +86 (871) 5920570.

# Supplementary Figures

**
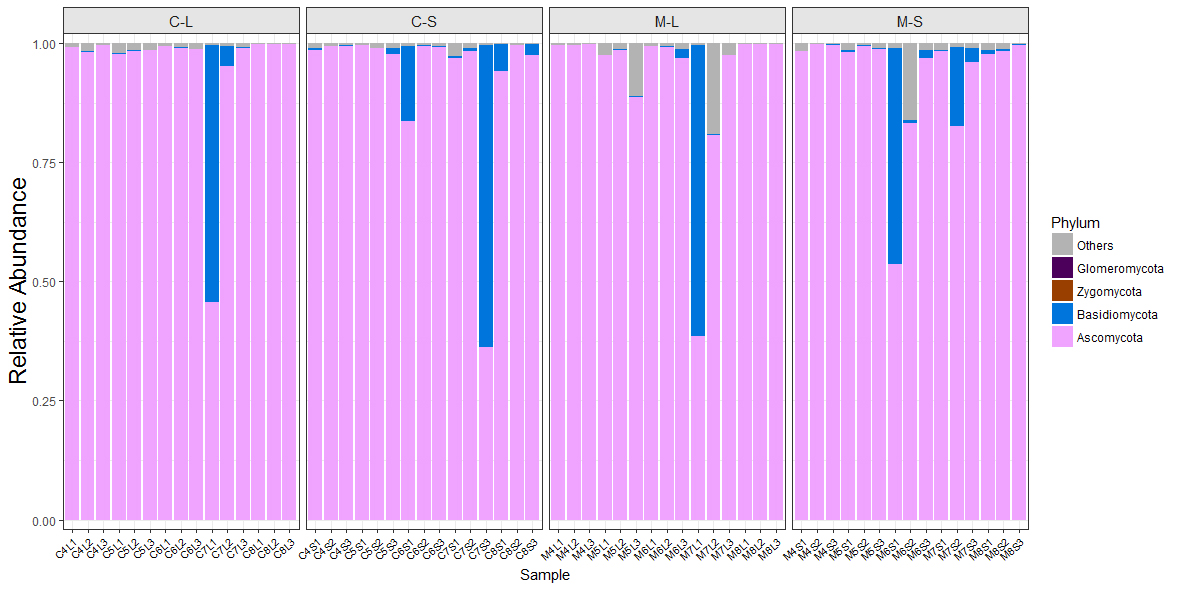
**

**FIGURE S1.** Relative abundance of endophytic fungi of two *Rosa* varieties at phylum-level. The fungi with a relative abundance below 0.1% were grouped as “others”. MS and ML, CS and CL: Stem and leaf of *R. multiflora* and *R. multiflora* var. *carnea*, respectively; 1, 2 and 3 represent triplicate. 4, 5, 6, 7 and 8 stand for April, May, June, July and August.

**FIGURE S2.** The relative abundance of *Neofusicoccum* at each developmental stage in the stems and leaves of two rose varieties. Error bars are standard error (n=3).

# Supplementary Tables

**TABLE S1** The Shannon index (*H’*) of two wild roses with different powdery mildew (PM) resistance

| Developmental stage | Shannon index (*H′*) | | | |
| --- | --- | --- | --- | --- |
|  | *R. multiflora* | | *R. multiflora* var*. carnea* | |
|  | Leaf | Stem | Leaf | Stem |
| April | 1.33±0.06^*^ | 1.21±0.22^**^ | 1.92±0.10^*^ | 1.72±0.06^**^ |
| May | 2.28±0.17 | 2.80±0.16 | 2.11±0.05 | 2.04±0.36 |
| June | 2.54±0.51 | 3.20±0.29 | 2.44±0.40 | 2.67±0.18 |
| July | 2.59±0.48 | 2.97±0.14 | 2.29±0.08 | 2.30±0.29 |
| August | 3.16±0.04 | 3.28±0.30 | 2.30±0.09 | 2.79±0.40 |

Data shown with Averages of replicates ±standard error; Asterisks indicate significantly higher values (* *p*< 0.05; ** *p*< 0.01); one-way ANOVA was used to analyze data from April, May, June, and July. If the mean difference between groups was statistically significant, post hoc pairwise test was made. Kruskal–Wallis test was used to analyze data from August (χ^2^ = 5.821, *p* > 0.05)

**TABLE S2** Effect of Variety, position and time on fungal communities revealed using PERMANOVA analysis

| Factor | Df | Sums of Sqs | Mean Sqs | *F* | *R^2^* | *P* |
| --- | --- | --- | --- | --- | --- | --- |
| Variety | 1 | 0.392 | 0.392 | 2.782 | 0.031 | 0.010^*^ |
| Tissue | 1 | 0.361 | 0.361 | 2.564 | 0.029 | 0.016^*^ |
| Developmental stage | 4 | 4.378 | 1.094 | 7.772 | 0.348 | 1e-04^***^ |
| Residuals | 53 | 7.464 | 0.141 | NA | 0.593 | NA |
| Total | 59 | 12.59 | NA | NA | 1 | NA |

* *p*<0.05, ****p*<0.001
